# Implementing change in primary care practices using electronic medical records: a conceptual framework

**DOI:** 10.1186/1748-5908-3-3

**Published:** 2008-01-16

**Authors:** Lynne S Nemeth, Chris Feifer, Gail W Stuart, Steven M Ornstein

**Affiliations:** 1 College of Nursing, Medical University of South Carolina, Charleston, South Carolina, USA; 2 Department of Family Medicine, University of Southern California, Los Angeles, California, USA; 3 Department of Family Medicine, Medical University of South Carolina, Charleston, South Carolina, USA

## Abstract

**Background:**

Implementing change in primary care is difficult, and little practical guidance is available to assist small primary care practices. Methods to structure care and develop new roles are often needed to implement an evidence-based practice that improves care. This study explored the process of change used to implement clinical guidelines for primary and secondary prevention of cardiovascular disease in primary care practices that used a common electronic medical record (EMR).

**Methods:**

Multiple conceptual frameworks informed the design of this study designed to explain the complex phenomena of implementing change in primary care practice. Qualitative methods were used to examine the processes of change that practice members used to implement the guidelines. Purposive sampling in eight primary care practices within the Practice Partner Research Network-Translating Researching into Practice (PPRNet-TRIP II) clinical trial yielded 28 staff members and clinicians who were interviewed regarding how change in practice occurred while implementing clinical guidelines for primary and secondary prevention of cardiovascular disease and strokes.

**Results:**

A conceptual framework for implementing clinical guidelines into primary care practice was developed through this research. Seven concepts and their relationships were modelled within this framework: leaders setting a vision with clear goals for staff to embrace; involving the team to enable the goals and vision for the practice to be achieved; enhancing communication systems to reinforce goals for patient care; developing the team to enable the staff to contribute toward practice improvement; taking small steps, encouraging practices' tests of small changes in practice; assimilating the electronic medical record to maximize clinical effectiveness, enhancing practices' use of the electronic tool they have invested in for patient care improvement; and providing feedback within a culture of improvement, leading to an iterative cycle of goal setting by leaders.

**Conclusion:**

This conceptual framework provides a mental model which can serve as a guide for practice leaders implementing clinical guidelines in primary care practice using electronic medical records. Using the concepts as implementation and evaluation criteria, program developers and teams can stimulate improvements in their practice settings. Investing in collaborative team development of clinicians and staff may enable the practice environment to be more adaptive to change and improvement.

## Background

Translating research into practice has been difficult to achieve by many health services leaders, despite tools such as benchmarks and clinical guidelines [1]. The result is 'underuse, overuse, and misuse' of healthcare interventions [2-5] and national concerns related to patient safety. Despite the large scientific knowledge base providing evidence for quality healthcare, much of it is not used [3,6]. Health care systems continue to provide care that is highly variable and fails to achieve sustainable change in practice patterns through the adoption and implementation of recognized best practices and evidence-based medicine [7]. Information technology that can guide care, support best practices, and enable measurement is often not yet implemented in many primary care practices. Where electronic medical record (EMR) tools are used, a learning curve poses a barrier for physicians on the path to quality improvement [8].

Implementing tools to use evidence as a basis for decision-making in clinical practice requires concerted actions by individual clinicians and leaders that are often considered beyond the scope of usual practice management. New approaches are needed to create clinical environments where people can easily implement new ideas, use research findings, adopt best practices, and improve clinical outcomes. Many researchers have identified facilitators and barriers to adopting a more evidence-based practice [9-13], and some have recommended that organizational culture may need to be changed [14-17]. Leaders can play a pivotal role, addressing characteristics of the practice environment affecting culture, thus influencing the responsiveness of the players to change.

Primary care practices are complex adaptive systems that cannot improve in a linear and prescribed manner [18-20]. The competing demands of the practice and inertia by clinicians must be considered when introducing improvements in the delivery of care [21,22]. Complexity science provides a lens that encourages local adaptation of processes to suit the needs of the practice members involved, yet the larger policy contexts that affect the care environment often require identification of a specific process for change to be successful [23].

This research explored the process of change used to implement clinical guidelines for prevention and treatment of cardiovascular disease and stroke within practices participating in the PPRNet-TRIP-II (Practice Partner Research Network-Translating Research into Practice) randomized clinical trial. PPRNet-TRIP-II tested the impact of quarterly performance reports, site visits, and network meetings on guideline adherence in primary care practices that use a common EMR tool [24]. The logic behind the intervention and the strategies used by practices to improve care [25,26] and the results of the clinical trial are reported elsewhere [27]. Study findings demonstrated performance improvements made by the practices, but did not explain how the practices accomplished meaningful change. The research reported here was designed as part of the process evaluation of the PPRNet-TRIP II study, to develop a theoretical framework explaining the process of change, so that more informed implementation and evaluation might be facilitated in future studies or demonstration projects.

## Methods

### Guiding framework

This study was guided by a number of pre-existing conceptual frameworks, most notably microsystems [28,29]. A microsystem is defined as a 'small organized patient care unit with a specific clinical purpose, set of patients, technologies, and practitioners who work directly with these patients'. Primary care practices are distinct clinical practice units with a designated purpose and function, fitting this definition well. Nine instrumental components of successful practices or clinical environments were previously identified within the Institute of Medicine's study on microsystems.

Microsystems are organized around four conceptual quadrants (each with instrumental components), including: Leading Organizations (clinical microsystem leadership, culture, organizational support); people (patient focus, staff focus, interdependence of the care team); performance and improvement (process improvement, performance patterns; and information (information and technology). Using microsystems as an overarching perspective within this research facilitated understanding how leadership functioned in each practice; the roles of the people working within the practice; the level of performance and investment in improvement; and the way information was handled, both at the technological and basic communication levels. This provided an organizational structure to examine the context of implementing change in practice. Microsystems guided a cultural assessment of the practice's implementation of change which focused on the relationships of the individuals involved, and the interdependence and effectiveness of the team.

Site visits to the practices enrolled in the intervention group of PPRN-TRIP-II created the opportunity for the lead author to directly observe practices in their natural environment and record field notes. Semi-structured interviews provided perceptions of staff and clinicians about each practice setting, including leadership and organizational characteristics. An integrated approach [30] to qualitative data analysis was used that incorporated inductive code generating, as well as a deductive organizing framework from the multiple theoretical perspectives that guided this research. A hermeneutical process of immersion and crystallization [31] confirmed the conceptual framework as an explanatory theory on the process of change. The institutional review board at Medical University of South Carolina approved this research.

### Sample and sampling strategy for the practice interviews

Eight primary care practices within the PPRNet-TRIP II intervention group participated in semi-structured interviews. The sample included small private internal medicine or family medicine practices that used a common EMR system (Practice Partner™, Seattle, WA), joined a practice-based research network (PPRNet), and agreed to participate in the parent study investigating quality improvement (QI) for primary and secondary prevention of cardiovascular disease and strokes. It is acknowledged that this may be an atypical sample of early adopters, yet this group of practices who were implementing changes in practice were in an ideal position to describe the challenges and opportunities inherent in the process. These primary care practices represented 'real-world' perspectives regarding the multiple changes taking place within the rapidly changing healthcare system.

Twenty-eight participants were selected (Table 1 provides characteristics of this sample) for the interviews which included office (managers, receptionists), clinical staff (nurses, medical assistants), and clinicians (physicians, nurse practitioners or physician assistants). A purposive sampling strategy was used to elicit a variety of reports about barriers to implementation and successful approaches to making change. A large variety of different perspectives was sought to prevent bias in the sampling process and to look for different views and possible discordance. In three solo practices and three practices with two clinicians, fewer individuals participated in the interviews, and where there were more clinicians (two practices) a greater number of interviews were conducted.

**Table 1:** Participants (pseudonyms) and Practices Represented

**Participant**	**Age Range**	**Gender**	**Ownership**	**Practice Role**
**Practice 1 *2 MDs***			**Region: Northeast urban**	

Alice	40–44	Female	Partner	Clinician/physician
Barry	45–49	Male	Owner	Clinician/physician
				
**Practice 2 *solo***			**Region: Southeast suburban**	

Carl	35–39	Male	Owner	Clinician/physician
Diane	45–49	Female	Employee	Clerical staff
Elaine	25–29	Female	Employee	Clinical support
				
**Practice 3 *solo***			**Region: Northwest suburban**	

Fran	55 or older	Female	Owner	Clinician/nurse practitioner
Gail	30–34	Female	Employee	Clerical staff
Hannah	45–49	Female	Employee	Clinical support
				
**Practice 4**	***>3 MDs***		**Region: Northwest large town**	

Ida	55 or older	Female	Employee	Clinical support
Jack	40–44	Male	Owner	Clinician/physician
Kathy	30–34	Female	Employee	Clerical staff
Linda	45–49	Female	Employee	Clinical support
Michael	40–44	Male	Partner	Clinician/physician
Nancy	50–54	Female	Employee	Clerical staff
				
**Practice 5**	***>3 MDs***		**Region: Northwest small town**	

Olive	45–49	Female	Employee	Clinician/physician assistant
Paula	45–49	Female	Employee	Clinical support
Rita	35–39	Female	Employee	Clerical staff
Sally	35–39	Female	Employee	Clerical staff
Tom	30–34	Male	Partner	Clinician/physician
				
**Practice 6**	***2 MDs***		**Region: Midwest rural**	

Uma	20–24	Female	Employee	Clinical support
Valerie	45–49	Female	Owner	Clinician/physician
Xena	45–49	Female	Employee	Clerical staff
Yolanda	40–44	Female	Employee	Clinical support
				
**Practice 7**	***2 MDs***		**Region: Midwest urban**	

Andrew	50–54	Male	Owner	Clinician/physician
Betty	50–54	Female	Partner	Clinician/physician
Zoe	30–34	Female	Employee	Clerical staff
				
**Practice 8**	***solo***		**Region: Southeast large town**	

Glenn	50–54	Male	Owner	Clinician/physician
Dana	20–24	Female	Employee	Clinical support

### Data sources

A semi-structured interview schedule was adapted from the Microsystems in Healthcare [28] study (Table 2). The interview explored the participants' interest in improvement and their own perceptions regarding enablers and barriers to that process. The questions were a starting point in the initial interviews; as the participants responded to these questions additional questions emerged, and were used within subsequent interviews. The lead author conducted all of the semi-structured interviews. Field notes were taken during the site visits that consisted of observations regarding the process of site visits, reactions of staff to academic detailing regarding cardiovascular prevention and treatment indicators within the PPRNet-TRIP II project, progress made on changes planned at prior visits, and new action plans of the practices reflecting their priorities.

**Table 2:** Semi-structured Interview Guide

**Level of Performance**	**Investment in Improvement**	**Leadership**
How successful do you feel you are (at the practice level) implementing change?	Describe what your system has done to implement the project, and improve quality.	Have there been any special efforts to develop an effective team?
How do you define success?	What specific strategies have you used to improve performance on selected indicators?	How does the leadership of this system affect the care that is provided here?
Describe the day-to day work environment of your system.	What assisted in making it successful?	How does the practice handle new ideas?
What are the communication patterns in the practice?	What have been the barriers?	Have new leaders (formal or informal) emerged to champion quality improvement efforts?
	How have these been overcome?	What is helpful?
		What does not assist in improving care here?

Legend: (adapted from Microsystems In Healthcare [28])

### Data collection and analysis

The interviews were recorded using an Olympus DS-330 digital voice recorder. Files were transcribed by an administrative assistant, verified by the primary investigator, and exported into NVivo 2.0 (QSR, Pty. Doncaster, Victoria, Australia) for coding.

Initial codes were developed using empiric sources from the literature about change management [32] and barriers to implementing guidelines [9], and an iterative process was used in the analysis that generated new codes as theoretical hunches emerged. Using constant comparison [33,34], codes were added, and then consolidated to the key themes that summarized the data. The transcripts were reviewed by three qualitative researchers and coding validated at both early and late stages in the analysis.

By reading aloud the transcripts of several practices with different experiences in the process of change and different levels of performance outcomes, immersion in the data by three qualitative researchers (LSN, CF, BFC) led to crystallization of key meanings (prompting questions and offering explanations that clarified and confirmed the framework that resulted from mapping the key concepts).

## Results

**Figure 1:**
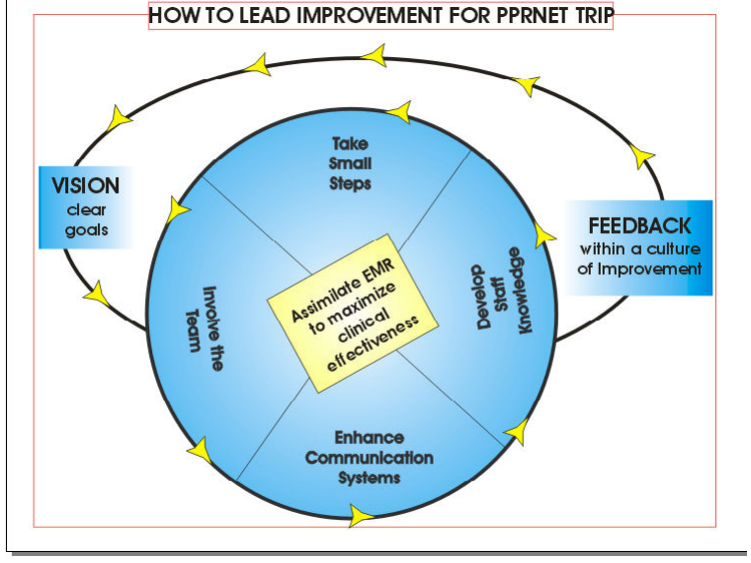
**How to Lead Improvement for PPRNet**. The concepts of the model reflect an iterative and interactive process by which additional cycles of change are stimulated through performance feedback and subsequent opportunities to modify vision with clear goals.

Through identification of the core themes, concepts and relationships, the framework was developed. Figure 1 provides an image representing the process of change that was undertaken by the practices. Clear leadership from the practice leaders was seen as an important component of the framework for implementing change in practice 'How to Lead Improvement for PPRNet-TRIP'. The following concepts elaborate the process of how change occurred within these practices:

1. Vision with clear goals

2. Team involvement

3. Enhance communication systems

4. Develop staff knowledge

5. Take small steps

6. Assimilate electronic medical record (EMR) into clinical practice to maximize clinical effectiveness

7. Feedback within a culture of improvement

The concept 'assimilating the EMR into clinical practice to maximize clinical effectiveness' was central to explaining how practices changed within PPRNet-TRIP II. The framework's focus on improvement and guideline implementation through better use and adaptation of processes within the practices' EMR tools is fitting for a group of practices engaged in a practice based research network (PBRN) aligned around a common EMR system. The forthcoming sections illustrate the concepts of the framework. The framework synthesizes the enabling strategies for change, which were present throughout the sample, but not necessarily seen in every practice. Pseudonyms were assigned to those interviewed, whose comments follow as the framework is explained.

### Vision with clear goals

Practices were most effective at change when the practice leader set a clear vision. In these practices, staff members discussed the goals for change. A physician in a solo practice who achieved significant change in practice performance benchmarks explained:

#### Dr. Carl

'It is defined in the guidelines, my professional responsibility for success. That is my profession: to get from point A to B. I use information that comes from the specialists in the studies that I am following, and that's how I gauge my success.'

Dr. Carl articulated what was important for him in his practice, that being successful in his patient care management was his responsibility as a physician. He established vision by determining which quality benchmarks were necessary for his practice to achieve, so his patients could benefit. His staff members understood the vision for his practice.

In another practice both physicians discussed the importance of vision and goals. The two physicians articulated a high regard for establishing goals and defining what needed to be accomplished.

#### Dr. Alice

'We look at the site visits as needs assessments to identify goals. It's important that you explain what the goals are, get buy in to work with people, and to see how you can help them to accomplish those things.'

#### Dr. Barry

'Our goal is to be like an old-fashioned family practice, with responsiveness on the same day as needed.'

### Involve the team

When staff members were clear about the vision and goals, felt included in decision-making, and were responsible for leading some component of the work plan to achieve results they adapted to make change happen. The nurse who worked with Dr. Glenn demonstrated effective teamwork through this comment.

#### Dana

'Basically to help Dr. Glenn I try to make his life simpler and make him go faster. I do a lot of the recalls, sending out the letters, to get a hold of the patients. When they are here, I make sure that they get everything done that they need, a little bit of everything, really ...Templates. I do a lot more with them now ... I try to get the notes done before he walks in. So he can do more talking with them instead of typing, and when he walks out he just has to put in his recommendations and impressions and then he'll be done with it all.'

A business manager of one practice related how well the team members work together.

#### Diane

'The biggest asset we have is our employees. We are like a well-oiled machine. Everyone knows what they're doing, and things get done. It works.'

This manager's perspective was that staff contributed to improved outcomes in patient care through teamwork. This practice valued clear leadership, working well together, and staff competence to do things the right way. This allowed them to be successful with their patients' care management. Observations in the practice environment revealed a cohesive group of staff who followed through with improvement goals they established.

#### Diane

'It's been a group effort. Everybody has to see the need. It's actually lives that you're saving; it's not just numbers ...just again, empowering the nurses, Dr. Carl has been real clear about use of guidelines, and to get the nurses and patients more involved in their care management. When patients call in for an appointment they are asked to plan for a cholesterol check. Continuing to call if they miss a visit, we stress the importance of these tests. It's leadership but it's also good patient care.'

A clerical staff member in a larger Northwest practice commented on what worked well and what did not regarding the involvement of the team.

#### Sally

'I think having a set of standards is really helpful, and the set criteria as to what needs to get done, and the goals. It still fluctuates some because we do have different personalities that do not necessarily agree. But everybody seems to know what the goals are and that really seems to help. What doesn't help is when one provider feels like they are being singled out because they are not doing it that way. And that kind of happens from time to time. I don't think that's real helpful.'

A physician leader in this practice discussed the new team approach:

#### Dr. Tom

'It's important for people to be honest and up front and have a level playing field with individuals talking to each other as professionals and not having a hierarchy where like the medical assistants don't talk to the doctors.'

### Enhance communication systems

Communication was enhanced by using the features of the EMR system more efficiently. Patient care needs were communicated within some practices using letter templates that reported results of diagnostic tests with therapeutic goals. Additionally, clinicians and staff used electronic mail within the EMR for internal messaging and reminder systems to help improve internal communication. One of the physicians discussed how patients are informed about when to follow-up regarding their laboratory tests:

#### Dr. Andrew

'... as part of our result letters we have the reminder put in about when they're supposed to get checked again.'

Clinicians followed-up on the important details of patient care through several embedded (within the EMR) communication systems. Dr. Betty explained the reminder systems for follow up with patients, and illustrated how practice members communicated effectively with each other.

#### Dr. Betty

'We communicate through our staff extensively. The facilitators for that communication are internal e-mail, and the EMR is huge in terms of inter-physician and staff ... to get things done... also in future activation we also use the e-mail ...send yourself one so that three weeks from now you remember to go back to X or Y or check on things. Then, we use the letters within Practice Partner to do a whole ton of communications to the patients, and the recall letters in the billing to activate patients to come in. Of course, we talk to each other face to face. And the staff talks to the patient by phone. We talk to the patient by phone. I would say [we use] every known strategy [of] communication, except e-mail. We studiously avoided e-mail ...for communication [with the patients].'

### Develop staff knowledge

While involving the team is an important concept, additional effort must be undertaken to develop knowledge (related to the clinical guidelines being implemented) of practice staff. Staff must understand the rationale for the work they are engaged in to be most effective. By providing avenues for staff to ask questions, office and clinical staff can provide critical reinforcement of the ideal plan of care and help the patients understand treatment goals and the importance of follow-up.

Dr. Glenn discussed how development of the staff occurs within his practice, which enabled the nurses to integrate their assessments into the templates that drive the patient care in his practice.

#### Dr. Glenn

'I have spent time to work closely with the nurses, to make the templates be very clear and effective to our practice... the nurses and I work very close together, and are real clear about what they need to do. The templates are developed together, to make things workable and make sense to the nurses since they do the data collection. It helps to make things work smoothly.'

The nurse in this practice expressed how she learns what is most important for patient care during dedicated time to develop templates and systems.

#### Dana

'We usually go over the templates and what we need for each disease process, what questions we need to ask. ...it cues us on what needs to be done when the patient is here. And [during these meetings we receive] just overall education on what it is we are trying to achieve, to let them [the patients] know where exactly we are trying to get to.'

### Take small steps

When making changes in practice, perfection is not needed to embrace a different approach. All of the practices had taken small steps, trying new methods and adjusting to the changes in their practice as they sought to embrace the clinical guidelines. Taking small steps implies motivation is present within the practice, and willingness to test a small change in practice.

Staff from the Northwest family practice discussed the small steps they had been taking in making changes in the practice that related to the guidelines. More was being delegated to the non-clinical staff in this practice to ensure patient communication and follow up was occurring as the practice decided. Clinical staff increased their efforts to use the EMR more actively than previously.

#### Ida

'We're seeing a lot more diabetics that are following up, you know, making sure they get done what they need every three months, every six months.'

#### Kathy

'The biggest change I've noticed were the letters going out, and then of course, the huge influx of patients and getting those patients in for their [glycosolated] hemoglobins and blood pressure checks.'

#### Linda

'As far as anything new with this project, the most different thing is that I note in the open chart note for diabetics, especially for lab work.'

### Assimilating the EMR into clinical practice to maximize clinical effectiveness

Using the EMR features more robustly assists with embedding evidence-based guidelines into practice. The practices and participants had different levels of expertise and experience with the use of the Practice Partner™ EMR system. Participants modified their approaches and methods to document in the record, search within the record, organize care, and use recalls for disease management.

#### Dr. Michael

'Well, I'd have to say that the physicians have definitely had to change the way they practiced. That's probably more in terms of utilization of the medical record. But that is what we probably should be doing anyway ...I can actually come and review my labs from the day before and then process the lab letter, which I can then give the staff and document in the back part of it. Actually it is working very well.'

### Feedback within a culture of improvement

Change in the practices was most enhanced by PPRNet-TRIP interventions. This had an impact on the practices' organization and communication. A culture of participation and a competitive spirit emerged among numerous practices within the intervention group, revealing the motivating effect of feedback from the intervention. Practices received performance data on the quality indicators quarterly. Dr. Valerie explained how dedicating time for prioritizing performance improvement within her practice was valuable:

#### Dr. Valerie

'I think the patients' achievements themselves give you the kind of day-to-day feedback that keeps you going ... I think that what I am doing differently now is what I thought I was doing before. I do a better job of it now. I have an understanding of how I can go about measuring the effectiveness of any particular approach that I am doing. And, it also has to do with the aging of the practice. I could have continued to emphasize care of younger people and health maintenance to a degree that would have eventually succeeded in excluding people who have chronic health problems cause they were going to move on or die...So, I think it just clarified in my mind that this is actually where the most effect is going to be felt.'

## Discussion

This research established a conceptual framework that explains how the process of change was perceived by small primary care practices that were implementing clinical guidelines and using a common EMR. The framework that was developed in this research can be used to establish a strategic plan for practice improvement that involves implementing EMR systems and clinical guidelines. The seven concepts (vision with clear goals; team involvement; enhance communication systems; develop staff knowledge; take small steps; assimilate electronic medical record (EMR) into clinical practice to maximize clinical effectiveness; and feedback within a culture of improvement) may catalyze action plans by similar practice teams that are ready to embark on improvement efforts. By focusing attention to these specific concepts inherent in the process of implementing change in primary care, leaders and practice members can become clearer about what they seek to change, why it is important, and how they can get there. A blueprint for implementing change, in the spirit of improvement and learning, can be developed by using these concepts. As an evaluation framework, each concept should be addressed and specific strategies formulated to engage the stakeholders in the process of change.

Microsystems [28,29] informed the design and analysis of this research, and led to a new framework for implementing change that elucidated seven concepts. Microsystems provided a mechanism to drill deeper into the meaning of the process of change, viewed from the perspective practice staff and clinicians implementing guidelines and improving quality with their EMR systems, as part of a practice-based research network. Within this study, as in the initial research on microsystems, qualitative findings about the behaviour, attitudes, and experience of small practice groups were helpful in explaining how the results of performance improvements were accomplished. Our study further refined the broader elements noted within the microsystems framework, by seeking the views of participants engaged in specific improvements. It further clarified the specific components related to making changes in practice related to implementing guidelines using EMRs. This study examined the microsystems concepts within small independent practices, unaffiliated with a larger health care system where the original concepts were identified.

Many of the concepts described within this research were noted in practices that were higher performers on the quality indicators and at accomplishing improvement from the baseline of the PPRNet-TRIP II study. Lower performing practices also demonstrated some elements of the model, but seemed to need more work to accomplish measurable improvement. More time to develop these strategies may be needed, as the practices become more receptive to quality improvement using benchmark data as feedback. Additionally, many practices need some time to develop the staff to adopt a higher level of responsibility in the practice.

### Comparisons and contrasts to previous research

Developing the clinical team in primary care practice is important to successfully implementing change in practice. In a case study of one exemplary primary care practice without an EMR, Solberg and colleagues [35] found that 12 principal attributes explained their excellent outcomes: visionary leadership; patient-centeredness; strong support for physician-patient relationship; strong group, team and standardization orientation; extensive involvement and management of all physicians and staff; highly organized change management; focused; strong change and improvement orientation; broad physician sense of ownership and responsibility; market driven; data-based, transparent and accountable; and pride and joy. This practice's culture of 'leadership and patient-centeredness' influenced core changes within the group to adopt team processes that focus on quality. Our findings validate several findings within Solberg's case study. We also noted the need for visionary leadership and further specified the need to set clear goals. We found that facilitating strong group, team orientation is enhanced through staff member involvement and staff development; and change and improvement orientation are energized by using the EMR more effectively and providing performance data feedback on improvement efforts. As practices became more transparent about their performance data, higher goals were continually developed as they reached a higher number of the performance targets in the parent study.

Crosson and colleagues found that in their case study of one practice that implementing an EMR without understanding how communication and decision-making occur, and how to resolve conflicts may undermine the benefits of the information system's potential to improve care [36]. Our finding that enhancing communication systems as a key component to developing a viable change process emphasizes the importance of this proactive component in the planning of change. Understanding the motivation of key stakeholders, resources and opportunities for change and outside motivators also is important. Cohen et al., found that change was influenced by complex interactions of factors inside and outside the practice [37]. Practice change occurred in relation to the interdependencies of: motivational reciprocity (systems that may motivate key stakeholders to make a change, and stakeholders who may motivate a change in systems); evaluating and exercising opportunities for change (helping stakeholders see opportunity for change); motivation, innovation, and independence (being realistic yet positive about opportunities for change); outside motivators and resources for change (being attuned to external forces); developing change trajectories (recognizing opportunities for change and paths to accomplish change); and external influences on the change option landscape (monitoring the external system and its impact on practice) . The emphasis in this model for change is on relationships and interdependencies. Taking Cohen's model for change further, we suggest additional work is needed to develop teams and staff knowledge regarding the guidelines being implemented.

By developing staff knowledge and translating guidelines into tangible steps that nurses, medical assistants, and office staff can embed into their practice patterns, changes in care delivery resulted in improvement in most practices. The exemplar quotes in the results section provide examples of how the involvement and development of team members result in assimilation of the EMR into the practice to maximize clinical effectiveness. Small steps towards new solutions were taken when practice leadership set the tone and direction within a practice. Change was implemented without long delays and procrastination for perfect solutions, when there was an ongoing source of feedback. The performance data that the TRIP-II practices received provided the measures that let practice teams know whether their newly implemented ideas were resulting in improvement.

Interest in developing teams to function at their highest level is not new, yet the evidence for this field is still in development. Interdisciplinary teams that balance input, participation, achievement, and openness to innovation perceive team effectiveness. [38]. Notably, nurses in primary care practices generally support clinical guidelines, and their role and influence within primary care is in a process of transition to one in which they may undertake responsibility for influencing the behaviour of clinicians [39].

#### Limitations and strengths of this research

The limitation of this research is that this research was conducted in a PBRN that involved self-selected practices interested in quality improvement and research in primary care practice, who were early adopters of EMR technology. Generalizability of these findings to unmotivated groups or groups without sufficient organizational resources may be limited; however the categories of strategies are generic. With similar practice characteristics, the framework might provide value towards implementing change. More work is needed to examine these concepts in other practices not affiliated with a practice-based research network, as well as in larger practices. The strength of this research is that it created an explanatory conceptual framework that could be used by similar practices to guide a change process. Using each of the concepts to create a blueprint for change, practice leaders may be able to engage staff to provide meaningful contributions to improving quality in primary care practice.

### Implications for future research

Interdisciplinary education has increased students' perceptions of professional roles [40-42]. Research is needed to evaluate the effectiveness of interventions for interdisciplinary continuing educational opportunities, and the relationship of such staff development on patient outcomes. Assuming a more team-oriented practice environment requires considerable investment in the education of staff within the setting. Structured approaches such as a quality team development program have promoted positive results in teamwork and patient outcomes [43]. Encouraging the staff to engage patients in appropriate ways that support and reinforce treatment goals may further enhance quality.

Activating learning cultures in primary care practice settings which encourage individual and team capabilities to learn together might stimulate aligned efforts to promote the patient's best interest. Cohesive vision can be developed together, based upon the complex system [44]. Further research is needed that evaluates the outcomes of interventions to promote 'learning practices'. This can strengthen the processes that interdisciplinary teams use to improve quality.

## Conclusion

A theoretical framework was developed to implement change in primary care practice that resulted from research within a group of small primary care practices.

Creating learning organizations is not an easy task for health care leaders, yet this direction is needed for the future and aligns well with the Future of Family Medicine's goals [45]. With practices adapted to effective teamwork, interdisciplinary learning and use of performance data to drive improvement leaders can shape more successful microsystems.

## Competing interests

This research was funded by Agency for Healthcare Research and Quality, US Department of Health and Human Services, Public Health Service. Grant No. 1 U18 HS11132-01. The authors declare they have no competing interests.

## Authors' contributions

LSN interviewed participants, coded the interview transcripts, analyzed the data and was principally responsible for the research idea, analysis and draft of the manuscript. CF reviewed all of the qualitative data, participated in the analysis and development of the framework, and editing of the manuscript. GWS provided leadership and direction to the first author in the research process, serving as the dissertation chair, and edited the manuscript. SMO was the principal investigator on the grant that funded this study, making this work possible. He provided oversight for this specific research within the context of the larger PPRNet-TRIP II study, enabling additional testing of these concepts within the research network. All authors reviewed and approved of the final manuscript.

## References

[B1] Kiefe CI, Allison JJ, Williams OD, Person SD, Weaver MT, Weissman NW (2001). Improving quality improvement using achievable benchmarks for physician feedback: A randomized controlled trial. JAMA.

[B2] Davis DA, Evans M, Jadad A, Perrier L, Rath D, Ryan D, Sibbald G, Straus S, Rappolt S, Wowk M, Zwarenstein M (2003). The case for knowledge translation: shortening the journey from evidence to effect. BMJ.

[B3] Chassin MR, Galvin RW (1998). The urgent need to improve health care quality. Institute of Medicine National Roundtable on Health Care Quality. JAMA.

[B4] Berwick DM (2002). A user's guide to the IOM's "Quality Chasm" report. Health Affairs.

[B5] McGlynn EA, Asch SM, Adams J, Keesey J, Hicks J, DeCristofaro A, Kerr EA (2003). The quality of health care delivered to adults in the United States. New England Journal of Medicine.

[B6] Berwick DM (2003). Disseminating innovations in health care.[comment]. JAMA.

[B7] Coye MJ (2001). No Toyotas in health care: Why medical care has not evolved to meet patients' needs. Health Affairs.

[B8] Miller RH, Sim I (2004). Physicians' use of electronic medical records: barriers and solutions. Health Affairs.

[B9] Cabana MD, Rand CS, Powe NR, Wu AW, Wilson MH, Abboud PAC, Rubin HR (1999). Why don't physicians follow clinical practice guidelines? A framework for improvement. JAMA.

[B10] Cabana MD, Ebel BE, Cooper-Patrick L, Powe NR, Rubin HR, Rand CS (2000). Barriers pediatricians face when using asthma practice guidelines. Arch Pediatr Adolesc Med.

[B11] Clark M (2003). Barriers to the implementation of clinical guidelines. Journal of Tissue Viability.

[B12] Cranney M, Warren E, Barton S, Gardner K (2001). Why do GPs not implement evidence-based guidelines? A descriptive study. Family Practice.

[B13] Jiang HJ, Lagasse RS, Ciccone K, Jakubowski MS, Kitain EM (2001). Factors influencing hospital implementation of acute pain management practice guidelines. Journal of Clinical Anesthesia.

[B14] Crawford P, Brown B, Anthony P, Hicks C (2002). Reluctant empiricists: community mental health nurses and the art of evidence-based praxis. Health & Social Care in the Community.

[B15] Lomas J (2003). Evidence-based practice in Steeltown: a good start on needed cultural change.[comment]. Healthcarepapers.

[B16] Porto JV (2003). A culture of expertise, not conflict.[comment]. Healthcarepapers.

[B17] Rundall TG, Shortell SM, Wang MC, Casalino L, Bodenheimer T, Gillies RR, Schmittdiel JA, Oswald N, Robinson JC (2002). As good as it gets? Chronic care management in nine leading US physician organisations. BMJ.

[B18] Miller WL, Crabtree BF, McDaniel R, Stange KC (1998). Understanding change in primary care practice using complexity theory.. Journal of Family Practice.

[B19] Miller WL, McDaniel R, Crabtree BF, Stange KC (2001). Practice jazz: Understanding variation in family practices using complexity science. Journal of Family Practice.

[B20] Crabtree BF (2003). Primary care practices are full of surprises!. Health Care Management Review.

[B21] Jaen CR, Stange KC, Nutting PA (1994). Competing demands of primary care: A model for the delivery of clinical preventive services. Journal of Family Practice.

[B22] Parchman ML, Pugh JA, Romero RL, Bowers KW (2007). Competing Demands or Clinical Inertia: The Case of Elevated Glycosylated Hemoglobin. Ann Fam Med.

[B23] Solberg LI (2000). Guideline implementation: What the literature doesn't tell us. Joint Commission Journal on Quality Improvement.

[B24] Ornstein SM (2001). Translating research into practice using electronic medical records the PPRNet-TRIP project: primary and secondary prevention of coronary heart disease and stroke. Top Health Inf Manage.

[B25] Feifer C, Ornstein SM (2004). Strategies for increasing adherence to clinical guidelines and improving patient outcomes in small primary care practices. Jt Comm J Qual Saf.

[B26] Feifer C, Ornstein SM, Jenkins RG, Wessell AM, Corley ST, Nemeth LS, Roylance LF, Nietert PJ, Liszka H (2006). The logic behind a multimethod intervention to improve adherence to clinical practice guidelines in a nationwide network of primary care practices. Evaluation & the Health Professions.

[B27] Ornstein S, Jenkins RG, Nietert PJ, Feifer C, Roylance LF, Nemeth L, Corley S, Dickerson L, Bradford WD, Litvin C (2004). A multimethod quality improvement intervention to improve preventive cardiovascular care: a cluster randomized trial.. Ann Intern Med.

[B28] Donaldson MS, Mohr JJ Exploring innovation and quality improvement in health care micro-systems: A cross-case analysis. http://www.nap.edu/catalog.php?record_id=10096#toc.

[B29] Nelson EC, Batalden PB, Huber TP, Mohr JJ, Godfrey MM, Headrick LA, Wasson JH (2002). Microsystems in health care: Part 1. Learning from high-performing front-line clinical units. Joint Commission Journal on Quality Improvement.

[B30] Bradley EH, Curry LA, Devers KJ (2007). Qualitative data analysis for  health services research: Developing taxonomy, themes and theory. Health Services Research.

[B31] Borkan J, Crabtree BF, Miller WL (1999). Immersion/Crystallization. Doing Qualitative Research.

[B32] Kotter JP (1996). Leading change.

[B33] Glaser BG (1992). Basics of grounded theory analysis.

[B34] Glaser BG (1998). Doing grounded theory: Issues and discussions.

[B35] Solberg LI, Hroscikoski MC, Sperl-Hillen JAM, Harper PG, Crabtree BF (2006). Transforming medical care: Case study of an exemplary, small medical group. Ann Fam Med.

[B36] Crosson JC, Stroebel C, Scott JG, Stello B, Crabtree BF (2005). Implementing an electronic medical record in a family medicine practice: Communication, decision making, and conflict. Ann Fam Med.

[B37] Cohen D, McDaniel R, Crabtree BF, Ruhe MC, Weyer SM, Tallia A, Miller WL, Goodwin MA, Nutting PA, Solberg LI, Zyzanski SJ, Jaen CR, Gilchrist VJ, Stange KC (2004). A practice change model for quality improvement in primary care practice. Journal of Healthcare Management.

[B38] Shortell SM, Marsteller JA, Lin M, Pearson ML, Wu SY, Mendel P, Cretin S, Rosen M (2004). The role of perceived team effectiveness in improving chronic illness care. Medical Care.

[B39] Harrison S, Dowswell G, Wright J (2002). Practice nurses and clinical guidelines in a changing primary care context: an empirical study. Journal of Advanced Nursing.

[B40] Fineberg IC, Wenger NS, Forrow L (2004). Interdisciplinary education: evaluation of a palliative care training intervention for pre-professionals. Academic Medicine.

[B41] Cooper H, Carlisle C, Gibbs T, Watkins C (2001). Developing an evidence base for interdisciplinary learning: a systematic review. Journal of Advanced Nursing.

[B42] Goodrow B, Olive KE, Behringer B, Kelley MJ, Bennard B, Grover S, Wachs J, Jones J (2001). The Community Partnerships Experience: a report of institutional transition at East Tennessee State University. Academic Medicine.

[B43] Macfarlane F, Greenhalgh T, Schofield T, Desombre T (2004). RCGP Quality Team Development programme: an illuminative evaluation. Quality & Safety in Health Care.

[B44] Rushmer R, Kelly D, Lough M, Wilkinson JE, Davies HTO (2004). Introducing the learning practice-II. Becoming a learning practice. Journal of Clinical Evaluation.

[B45] Future of Family Medicine Project Leadership Committee (2004). The future of family medicine: A collaborative project of the family medicine community. Annals of Family Medicine.

